# Enteral administration of bacteria fermented formula in newborn piglets: A high fidelity model for necrotizing enterocolitis (NEC)

**DOI:** 10.1371/journal.pone.0201172

**Published:** 2018-07-23

**Authors:** Shreyas K. Roy, Qinghe Meng, Benjamin D. Sadowitz, Michaela Kollisch-Singule, Natesh Yepuri, Joshua Satalin, Louis A. Gatto, Gary F. Nieman, Robert N. Cooney, David Clark

**Affiliations:** 1 SUNY Upstate Medical University, Department of Surgery, Syracuse, New York, United States of America; 2 SUNY Cortland, Department of Biology, Cortland, New York, United States of America; 3 Albany Medical Center, Department of Pediatrics, Albany, New York, United States of America; Gaziosmanpasa University, TURKEY

## Abstract

**Objective:**

To develop an animal model which replicates neonatal NEC and characterizes the importance of bacterial fermentation of formula and short chain fatty acids (SCFAs) in its pathogenesis.

**Background:**

NEC is a severe form of intestinal inflammation in preterm neonates and current models do not reproduce the human condition.

**Methods:**

Three groups of newborn piglets: Formula alone (FO), Bacteria alone (*E*.*coli*: BO) and *E*.*coli*-fermented formula (FF) were anesthetized, instrumented and underwent post-pyloric injection of formula, bacteria or fermented-formula. SCFA levels were measured by gas chromatography-mass spectrometry. At 6 h bowel appearance was assessed, histologic and molecular analysis of intestine were performed. Gut inflammation (p65 NF-κB, TLR4, TNF-α, IL-1β), apoptosis (cleaved caspase-3, BAX, apoptosis) and tight junction proteins (claudin-2, occludin) were measured.

**Results:**

SCFAs were increased in FF. Small bowel from FF piglet’s demonstrated inflammation, coagulative necrosis and pneumatosis resembling human NEC. Histologic gut injury (injury score, mast cell activation) were increased by Bacteria, but more severe in FF piglets. Intestinal expression of p65 NF-κB, NF-κB activation, TNF-α and IL-1β were increased in BO and markedly increased in the FF group (P<0.05 vs. FO). Intestine from Bacteria piglets demonstrated increased apoptotic index, pro-apoptotic protein expression and decreased tight junction proteins. These changes were more severe in FF piglets.

**Conclusions:**

Our piglet model demonstrates the findings of NEC in human neonates: systemic acidosis, intestinal inflammation, pneumatosis and portal venous gas. Bacteria alone can initiate intestinal inflammation, injury and apoptosis, but bacterial fermentation of formula generates SCFAs which contribute to the pathogenesis of NEC.

## Introduction

Necrotizing enterocolitis is an inflammatory disorder of the intestine affecting premature infants. The prevalence of NEC is 5–7% in very low birth weight infants [1] affecting approximately 3000 infants per year in the United States with associated mortality of 20–30% [2]. The major risk factors for NEC are prematurity, bacterial colonization, and formula feeding [3]. Most small animal models of NEC use hypoxemia and hypothermia to produce gut inflammation [4–8]. However, the role of asphyxia and hypothermia in human NEC is unclear [1, 3, 9–13]. Furthermore, these models do not completely reproduce the phenotype of neonatal NEC, which includes pneumatosis intestinalis, portal venous gas and intestinal inflammation [7, 14].

Increased expression of Toll-like receptor 4 (TLR4) in the premature intestine is an important factor in the pathogenesis of NEC [15]. Activation of intestinal TLR4 by gram negative bacteria in the neonatal gut recapitulates many aspects of NEC including intestinal inflammation characterized by intestinal epithelial apoptosis, activation of nuclear factor-κB (NF-κB) and inflammatory cytokine production [15]. Although bacterial colonization of the gastrointestinal tract is critical in the pathogenesis of NEC, efforts to identify specific bacterial pathogens responsible for causing NEC have been unsuccessful [16, 17]. Consequently, the treatment of NEC which includes cessation of feeding, broad spectrum antibiotics and surgical resection when complications occur has not changed for many years [3]. Despite many advances, the pathogenesis of NEC remains incompletely defined and currently available animal models of NEC are unable to completely reproduce the clinical, gross, and histopathological features of the disease [3, 18]. This gap in knowledge must be addressed to develop new therapies which prevent and/or effectively treat NEC.

The current study describes a piglet model of NEC which reproduces many features of neonatal NEC. We hypothesize bacterial fermentation of formula produces SCFAs which are important in the pathogenesis of NEC. Several studies suggest bacterial fermentation of non-hydrolyzed lactose is a consequence of immature intestinal lactase in preterm infants, leading to the production of SCFAs which are associated with the onset of intestinal lesions [19–21]. Di Lorenzo et al created a NEC model in neonatal piglets by injecting isosmolar acidified bovine alpha-casein, which amplifies the inflammation initiated by SCFAs [14]. To test our hypothesis we examine the relative importance of bacterial fermentation of formula vs. bacteria alone in the pathogenesis of NEC.

We used newborn female piglets because their gastrointestinal tract closely resembles human intestine. Our study compares the effects of enteral administration of bacteria-fermented formula, bacteria, and formula alone on gross and histologic intestinal appearance and molecular indicators of intestinal inflammation (TLR4 expression, NF-κB activation, inflammatory cytokine production, mast cell activation), enterocyte apoptosis and barrier function (claudin-2, occludin expression). We also perform *in vitro* studies of FF and SCFAs on neonatal intestinal epithelial cells (IECs) to assess their ability to directly cause epithelial inflammation. Collectively our findings support our hypothesis regarding the role of SCFAs in the pathogenesis of NEC.

## Methods

Anesthesia in newborn female Yorkshire was induced with glycopyrrolate (0.01 mg/kg, intramuscular), Thiopental (10 mg/kg IV), Telazol (tiletamine hydrochloride and zolazepam hydrochloride (5 mg /kg, intramuscular), and xylazine (2 mg/kg, intramuscular). Anesthesia was maintained by continuous infusion with Ketamine (3 mg/1ml) plus xylazine (0.003 mg/1ml). After ensuring adequate anesthesia by assessing eye reflex and pain reflex to footpad pinch, an open tracheostomy was performed and animals were connected to a G5™ ventilator (Hamilton Medical, Reno, NV) on volume cycled ventilation with tidal volume of 10 ml/kg, respiratory rate (RR) of 45/minute titrated to maintain PaCO_2_ within normal range with FiO_2_ of 30% and positive end-expiratory pressure of 3 cmH_2_0 throughout the 6 hour experiment. Piglets were fully anesthetized and monitored continuously throughout the 6-hour study period. Each piglet will be continuously monitored for six hours until euthanasia with pentobarbital (150 mg/kg). All of the animal experiments in this study were approved by the institutional animal care and use committee at Upstate Medical University (IACUC #: 176).

### Preparation of formula, bacteria, fermented formula and measurement of SCFAs

The infant formula was purchased from Enfamil (Glenview, IL). Non-toxigenic *E*. *coli* isolated from the stools of human neonates with NEC was provided by Dr. Clark (Albany Medical Center, Albany, NY) and was cultured according to standard protocols. *E*. *coli* is isolated in blood cultures from 30% patients with NEC [22, 23] and associations of *E*. *coli* with NEC have been previously reported [24, 25]. Briefly, a single colony of *E*. *coli* was picked from sheep blood agar plates, transferred to a culture tube containing tryptic soy broth (TSB) medium and incubated for 18 hours at 37°C. The bacteria were collected by centrifugation at 4500 rpm x 30 min, then re-suspended in TSB to quantify the number of bacterial cells by spectrophotometer at OD 600 nm. (A): Fermented Formula was prepared by inoculating 50 ml of Enfamil with 10^4^ *E*. *coli* and incubating for 18 h at 37°C. The pH of FF was tested using a pH probe to confirm the pH of 5 ± 0.5. (B): The bacterial solution was prepared by inoculating 50 ml of TSB with 10^4^ of *E*. *coli* and incubating for 18 h at 37°C. The bacterial pellet was re-suspended in normal saline (NS) solution (pH 5.5). A bacterial titer of 10^9^ CFU in the fermented formula and bacterial preparations was determined prior to infusion using a combination of spectrophotometry at OD 600 nm and back plating serial dilutions.

Filtered fermented formula was obtained by purification with a 0.45 microns filter. SCFAs (acetic, propionic and butyric acid) were measured in formula, resuspended *E*. *coli* and *E*. *coli*-fermented formula by Health Sciences (Bristol, CT) using a standard GC-MS analysis protocol.

### Animal experimental design, rationale, anesthesia and surgical procedure

Weaning Yorkshire piglets (Keystone Mills, Romulus, NY) one week of age were chosen for use in our experiment since its gastrointestinal tract structurally and physiologically resembles that of human neonates [26]. The gastrointestinal maturity in a preterm pig born at day 105 (full term = 116 d) resembles a preterm infant with a gestational age of 28–30 weeks [26]. 123 d old piglets were used in our experiment as sterile and immunologically virgin condition in the neonate could make the bacterial colonization and its responses inconsistent and unpredictable. In order to avoid the potentially confounding effects of gender, only female Yorkshire piglets were used [27, 28].

Piglets were anesthetized and monitored continuously throughout the 6-hour study period. Animals underwent tracheostomy for mechanical ventilation, central venous catheter for fluid resuscitation, and cystostomy for urine output monitoring. In brief, under aseptic conditions a midline laparotomy was made and gastrostomy (opening of stomach) was performed. Silastic catheters were inserted through the gastrostomy, advanced past the pylorus and the balloon was inflated to occlude the duodenal lumen to prevent egression of luminal contents. Next, the bowel was gently flushed with warm saline through the catheter in order to ensure a reduction of intraluminal bacterial load. Next, Formula, Bacteria or Fermented Formula were infused into the duodenum. Animals were managed using NICU standards of care. Animals were euthanized with pentobarbital (150 mg/kg) 6 hours after duodenal infusion. The entire small bowel and blood samples were collected. Segments of the proximal, middle, and distal small bowel were sectioned, fixed in formalin by immersion and frozen in liquid nitrogen. All of the animal experiments in this study were approved by the institutional animal care and use committee at Upstate Medical University (IACUC #: 176).

Animals were randomized to three groups: A. Formula only (FO, n = 5); B. Bacteria only (BO, n = 8): and C. Fermented Formula (FF, n = 7).

### Measurements

Temperature (°C), heart rate (HR: bpm), mean arterial pressure (MAP: mmHg), fluid infusion volume (ml/h), and urine output (ml/h) were measured hourly. Arterial blood gases and chemistries were measured hourly with Roche Blood gas analyzer (Cobas b221, Basel, Switzerland).

We developed a quantitative gross pathological score for NEC using the most common gross pathologic features of NEC: hemorrhagic inflammation of the bowel (congested, bloody, edematous inflammation); coagulative transmural necrosis (gray devitalized bowel tissue); intestinal perforation; pneumatosis intestinalis (visible bubbles beneath the serosa) and portal venous gas (visible bubbles in portal vein) [6, 11, 13, 29–32]. Each gross feature of NEC was scored as 0 if absent, 1 if present and 2 if severe. Scoring was performed by a single individual and was non-blinded since it was performed during necropsy and treatment groups were readily apparent.

Histologic analyses were performed with light microscopy using H&E stained sections. Quantitative analysis of histologic intestinal injury was performed using a validated scoring system [33]. The lengths of villi were measured (micrometers) from the villus tip to the crypt neck on H&E stained sections. Giemsa staining was used to distinguish activated mast cells. The number of activated mast cells (purple violet) per high-power field was assessed in the villi of intestinal tissue specimens. The percentage of activated mast cells over total mast cells was calculated to quantify mast cell activation. The scoring and counting were performed by a histologist blinded to treatment groups.

### RNA isolation and quantification

Total RNA was isolated from frozen intestinal homogenate using Trizol reagent (Invitrogen, Carlsbad, CA) according to manufacturer’s instructions. Amplification of cDNA was carried out by using iQ SYBER Green Supermix PCR Master Mix (BIO-RAD, Hercules, CA). The primers used for quantitative PCR analysis were: NF-κB forward, 5'-AAAGACTGCCGGGATGGCTTCTAT-3', reverse, 5'-TTCCAGGTCCCGCTTCTTTACACA-3; β-Actin forward, 5'-GGGAGATCGTGCGGGACAT-3', reverse, 5'-AGCACCGTGTTGGCGTAGAG-3'. β-Actin was used as the internal control. The target mRNA abundance was detected and normalized to that of β-Actin mRNA.

### TUNEL assay

To detect apoptotic intestinal cells, the TUNEL kit (Roche, Indianapolis, IN) was used according to manufacturer’s instructions. Sections were examined microscopically with localization of TUNEL positive nuclei relative to all nuclei from five randomly selected consecutive fields at 400x magnification by two blinded, experienced investigators. Apoptotic index was calculated as the number of TUNEL positive cells expressed as percentage of total cells.

### Western blot

Frozen small intestinal tissues were homogenized and lysed on ice in protein lysis buffer. Protein samples (20μg) were denatured with sample loading buffer and subjected to 12% SDS-PAGE gel, and then transferred to PVDF membranes. TLR-4 (sc-10741), NF-κB (sc-109; Santa Cruz) and BAX (sc-526; Santa Cruz), occludin (40-470-0 Invitrogen) and claudin-2 (32-560-0; Invitrogen), cleaved caspase-3 (9662; Cell Signaling Technology) and secondary HRP-conjugated antibody (Bio-Rad, Hercules, CA) were used. Bands were stained using Pierce ECL Western Blotting detection solution (Thermo Scientific, Rockford, IL) according to the manufacturer’s instructions and recorded by exposure of the membrane to x-ray film. Membranes were stripped and re-probed with GAPDH (Santa Cruz) to verify equal protein loading. Band intensity was quantified using ImageJ software (NIH). Immunoblot results were reported as relative densitometry units (RDU) normalized to GAPDH.

### Electrophoretic mobility shift assay (EMSA)

Nuclear extracts were obtained using Nuclear Extraction Kit (EMD Millipore) according to manufacturer’s instruction. Consensus oligonucleotides specific for NF-κB binding (5’-AGTTGAGGGGACTTTCCCAGG-3’) were end-labeled with ^32^P-ATP using T4 polynucleotide kinase (Promega, Madison, WI). 50μg of nuclear extracts were incubated with labeled consensus and binding buffer. NF-κB protein-DNA complexes and free ^32^P oligonucleotides were separated by gel electrophoresis. The gels were then dried and autoradiographed at -70°C. The intensity of DNA/protein complex bands were measured using Image J software.

### Cell culture, transfection and treatment

FHs 74 Int human neonatal intestinal epithelial cells were purchased from ATCC (Manassas, VA). The cells were routinely maintained in Hybri-Care medium (Cat. 46-X; ATCC) contain 10% FBS, 1% of streptomycin and penicillin (Cat. 15240062; Gibco), and 30 ng/ml EGF (Cat. z00333-1; GeneScript). Transfections were carried out using the Lipofectin 2000 (Invitrogen). The conditions of transfection of NF-κB expression vector were optimized (plasmid concentrations, transfection reagent concentration over time, etc.) in the beginning of experiments. Empty vector (pCMV4) was used as a control in all experiments. The transfected cells were washed three time before treatment and incubated in serum-free Hybri-Care media with filtered fermented formula (0–400 μl/ml) or propionic acid (0–5 mM) (cat. A258-500; Fisher scientific) for 18 h.

### Statistical methods

Endpoints were summarized using mean ± SE or median with interquartile range (IQR) depending on the distribution of the data. Differences among groups were assessed using either analysis of variance (ANOVA), evaluated for model fit using graphical analysis of the residuals, followed by pairwise comparison corrected using the Bonferroni method or the Kruskal-Wallis test followed by Dunn's multiple comparison method, again depending on the distribution of the data. Student’s t-test also was used to compare the differences between two groups. Statistical significance was declared at the 0.05 level. Box and whisker plots show: Mean (Solid Square), 25–75% range (open box), median (line across open box), minimum and maximum values (whiskers).

Statistical analysis of the data was performed using Prism 5.0 (GraphPad Software, San Diego, CA) and SPSS 11.5 (IBM SPSS Inc., Chicago, IL). A summary of the statistical tests used to analyze our data in this manuscript paper is provided in Table 1. A summary of experimental data and analysis of the methods used in this manuscript is provided as supplementary file (S1 Table).

**Table 1:** Data analysis.

	Group	Median	25%-75% percentile	N	P-value *
Fig 2D: Histological Score	FF	3.6	2.8–4.6	7	0.0002 *
	BO	0.4	0.29–0.46	8	
	FO	0		5	
Fig 7B: Mast cell count	FF	72.5	54.4–90.7	7	0.0002 *
	BO	41.1	30.9–51.4	8	
	FO	24.9	18.7–31.1	5	
Table 2: Acidosis (PH) At 6 h	FF	7.03	6.99–7.03	7	<0.0013 *
	BO	7.37	7.39–7.41	8	
	FO	7.37	7.37–7.4	5	
Table 2: Platelet at 6 h	FF	270	215–305	7	0.0015 *
	BO	410	378–435	8	
	FO	387	348–464	5	
		Mean	SD	N	
Fig 2E: Villus length	FF	18.17	3.66	7	<0.0007 *
	BO	31.66	4.37	8	
	FO	37.76	4.65	5	
Fig 3A: TLR4 protein	FF	0.84	0.11	7	<0.0001 **
	BO	0.71	0.12	8	
	FO	0.27	0.07	5	
Fig 3B: NF-κB mRNA	FF	1.14	0.39	7	<0.007 **
	BO	1.00	0.21	8	
	FO	0.57	0.12	5	
Fig 3C: NF-κB P65 protein	FF	0.76	0.18	7	<0.001 **
	BO	0.61	0.12	8	
	FO	0.38	0.10	5	
Fig 3D: NF-κB DNA binding activity	FF	4.92	0.63	7	<0.0003 **
	BO	2.81	0.24	8	
	FO	1.65	0.99	5	
Fig 4A: IL-1β levels	FF	9.05	5.75	7	<0.0001 **
	BO	8.47	3.07	8	
	FO	0.69	0.27	5	
Fig 4B: TNF-α levels	FF	1.04	0.63	7	<0.0081 *
	BO	0.89	0.14	8	
	FO	0.30	0.16	5	
Fig 5B:Apoptotic index	FF	42.2	4.26	7	<0.0001**
	BO	28.0	4.02	8	
	FO	14.7	2.41	5	
Fig 5C:Cleaved caspase-3	FF	1.39	0.24	7	<0.0004**
	BO	0.99	0.32	8	
	FO	0.57	0.26	5	
Fig 5D: BAX protein	FF	2.05	0.32	7	<0.0001 **
	BO	1.42	0.17	8	
	FO	1.16	0.17	5	
Fig 6A: Claudin-2 protein	FF	0.27	0.12	7	<0.0001 **
	BO	0.80	0.21	8	
	FO	1.14	0.28	5	
Fig 6B: Occludin protein	FF	0.55	0.14	7	<0.0001 **
	BO	1.04	0.27	8	
	FO	1.66	0.21	5	
Fig 8A: NF-κB activation	Control	1	0.18	4	0.0019 ***
	FF	2.37	0.48	4	
Fig 8B: NF-κB activation	Control	1	0.74	4	0.0017 ***
	Propionic acid	8.39	2.64	4	
Table 2: Fluid (ml) at 6 h	FF	123	42.0	7	0.003 **
	BO	48.0	45.0	8	
	FO	26.0	39.0	5	

**Notes**:

*: Kruskal-Wallis test and post Dunn's Multiple Comparison Test.

**: One-way ANOVA and post Bonferroni's Multiple Comparison Test.

***: t-test

## Results

The concentration of SCFAs were measured in samples of enterally administered Formula (n = 3), Bacteria (n = 4) and Fermented formula (n = 5). All three SCFAs were undetectable in Formula. Butyric acid levels were < 9.91 μg/ml in Bacteria and Fermented formula preparations. Propionic acid levels were < 10 μg/ml in Bacteria and 36.68 ± 4.83 μg/ml in Fermented formula. The concentration of acetic acid was 20.73 ± 0.59 μg/ml in Bacteria and 1783.82± 43.61 μg/ml in Fermented formula. These data show Fermented formula contains high levels of SCFAs, especially acetic and propionic compared to the Bacteria and Formula groups.

Baseline measurements are similar between groups (Table 2) and most parameters (HR, Temperature, MAP) did not change significantly over the study period (p>0.05). All three groups required fluid resuscitation to maintain MAP and urine output. The FF group had the greatest fluid requirements (123±42 ml/h vs 48±45 ml/h and 26±39 ml/h in the BO and FO groups respectively; p<0.01). Consistent with the need for more fluid resuscitation, the FF group developed a metabolic acidosis (p<0.05 vs. BO and FO) and a reduction in platelet count from 557,000/μL to 255,400/μL at 6 h (p<0.01 vs. BO and FO).

**Table 2:** Physiologic, metabolic, and blood parameters.

Parameters	Time	FF	BO	FO
HR (bpm)	BL	129.60 ± 5.6	144.8 ± 6.2	147.6 ±14.6
	6 h	95.6 ± 5.8	95.0 ± 11.0	91.4 ± 6.5
Temperature (°C)	BL	37.1 ± 0.3	36.1 ± 0.7	36.1 ± 0.3
	6 h	37.0 ± 0.3	36.9 ± 0.3	36.7 ± 0.4
MAP (mm Hg)	BL	85.1 ± 4.7	84.6 ± 10.4	91.0 ± 4.9
	6 h	72.9 ± 3.5	83.1 ± 8.8	92.8 ± 8.2
Fluid (ml)	6 h	123.0 ± 42.0^a^*	48 ±45.0	26.0 ± 39.0
Acidosis (PH)	6 h	7.0 ± 0.02^b^*	7.4 ± 0.04	7.4 ± 0.03
Platelet (Thousand per ml K/ml)	BL	557.0 ± 101.0	529.0 ± 120.0	522.2 ± 177.0
	6 h	255.4 ± 47.3^c^*	403.0 ± 34.0	402.0 ± 62.3

**Notes**:

*: FF vs. BO and FO;

**a**: One-way ANOVA and post Bonferroni's Multiple Comparison Test (p = 0.003);

**b**: Kruskal-Wallis test and post Dunn's Multiple Comparison Test(p:<0.0013);

**c**: Kruskal-Wallis test and post Dunn's Multiple Comparison Test (p = 0.0015).

**Fig 1:**
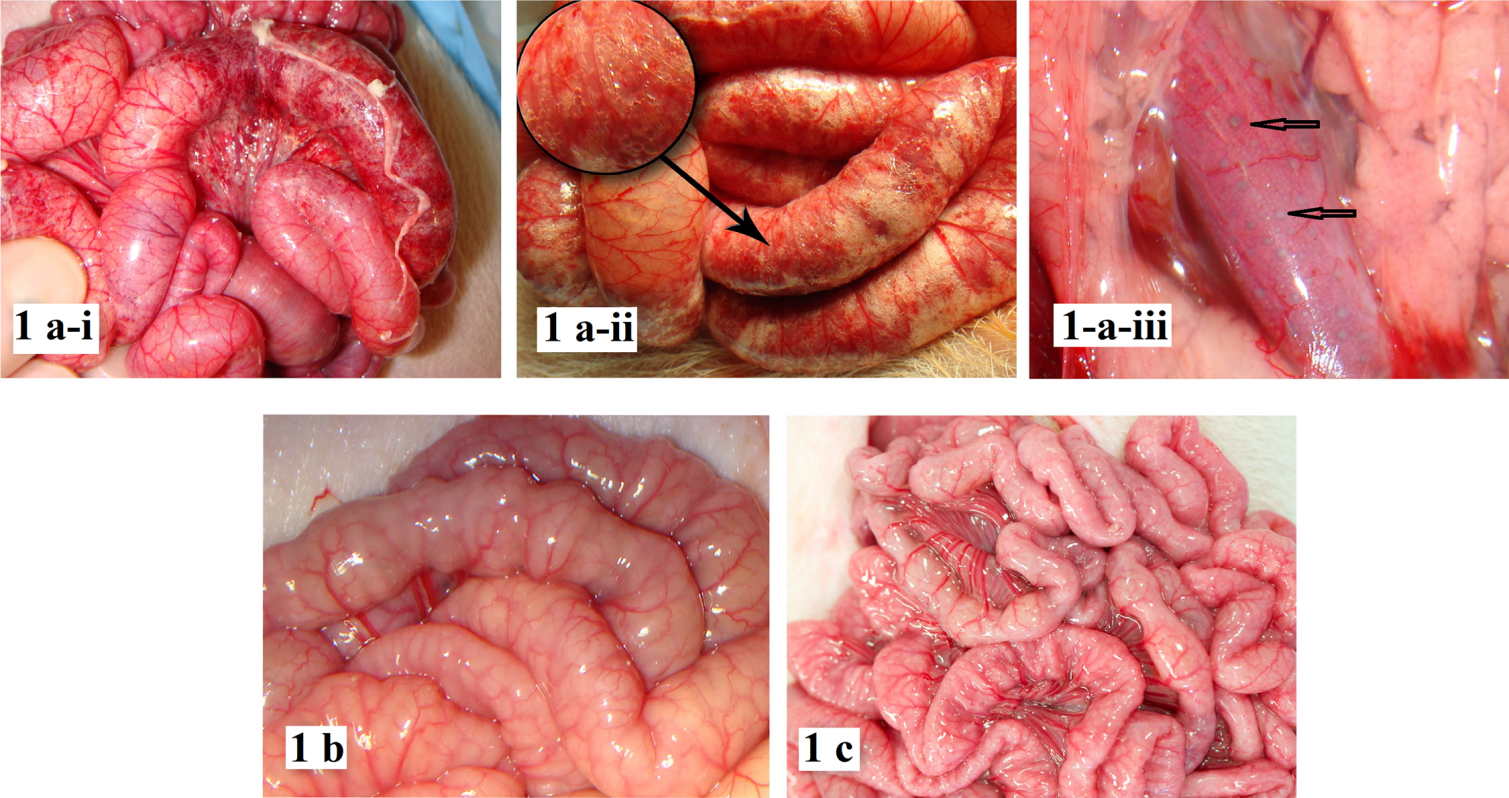
Gross pathology from fermented formula (FF), bacteria only (BO) and formula only (FO) groups. Fig 1A. Gross pathology from FF group: 1a-i. Small intestinal inflammation. Representative sample of small intestine from FF group animal fed with a mixture of nontoxigenic *E*. *coli* and standard infant formula. The bowel shows evidence of severe inflammation, transmural necrosis, and bowel wall edema, discontinuous “skip lesions. 1a-ii. Pneumatosis intestinalis. Representative sample of small intestine from FF group animal. Clear demonstration of gas bubbles in the sub-serosa of the small intestine confirming pneumatosis intestinalis. 1a- iii. Portal venous gas. Representative dissection of pancreas and portal venous tributary in FF group animal. Pancreatic parenchyma was retracted to demonstrate gas bubbles in the portal venous system confirming portal venous gas. Fig 1B. Gross pathology from BO group. Representative image of small intestine of an animal fed only E. coli suspended in normal saline. This mixture has an equivalent PH as the formula + bacteria mixture (PH = 5.5). The bowels are pink, healthy, and show no evidence of inflammation. Fig 1C: Gross pathology from FO group. Representative image of small intestine of an animal fed only infant formula, the bowels are pink, healthy, and show no evidence of inflammation. **Fig 2.** Histology from Fermented Formula (FF), Bacteria only (BO) and Formula only (FO) groups.

At necropsy, the FF group had the classic findings of severe neonatal NEC including hemorrhagic intestinal inflammation, coagulative transmural necrosis, intestinal perforation (Fig 1A-i) pneumatosis intestinalis (Fig 1A-ii) and portal venous gas (Fig 1A-iii). In contrast, the BO Fig 1B) and FO (Fig 1C) groups had grossly normal bowel on inspection with no signs of intestinal inflammation.

**Fig 2:**
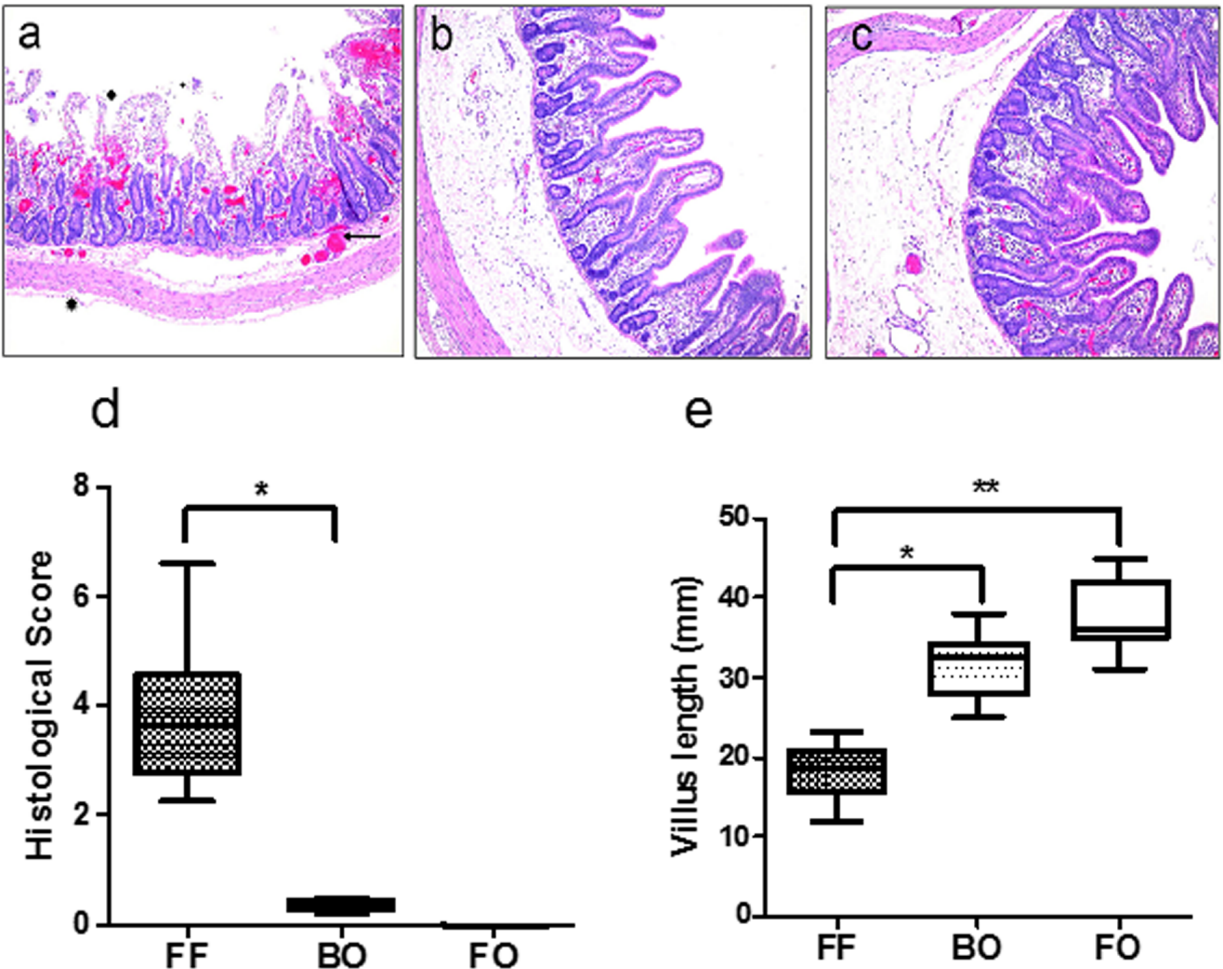
Fig 2A: Histology from FF group. Representative hematoxylin and eosin stained section of small intestine with classic features of NEC: Regional villus disruption & destruction of villus architecture, mucosal sloughing, blood congestion, separation of the sub-mucosa and lamina propria (pneumatosis intestinalis). Fig 2B: Histology from BO group. Representative section of small bowel showing no inflammation, normal villus anatomy and architecture. Fig 2C: Histology from FO group. Representative section of small bowel showing no inflammation, normal villus anatomy and architecture. Fig 2D: Histological Scoring in FF, BO and FO groups. Histogram of quantitative histological scoring of small intestine for each experimental group. Blinded quantitative scoring was performed as described in the methods. The histopathologic features of NEC (villous destruction, inflammatory infiltrates, and pneumatosis intestinalis) were assigned a quantitative score of zero if absent and 1 if present in a single microscopic field of 10x magnification. All animals in the FF group scored 4 in all sections analyzed, representing severe disease. BO & FO groups had normal intestinal histology. Histological scores from FF group were significantly higher than BO and FO groups. The data are represented as median and IQR (*P<0.05, **P<0.01, N = 5–8 / group). Fig 2E: Villus length in FF, BO and FO groups. The measurement of villus length was performed as described in the methods. The significant reduction in FF group were observed compared to BO and FO groups. The data are represented as median and IQR (* P<0.05, N = 5–8 / group).

The histological features of NEC are clearly demonstrated in a representative section of piglet bowel from the FF group (Fig 2A). These include extensive, complete destruction of villi architecture, blood congestion, transmural necrosis, and separation of the submucosa from the lamina propria, correlating histologically to pneumatosis intestinalis. In contrast, neither the BO (Fig 2B) nor FO (Fig 2C) groups had evidence of significant mucosal damage. Quantitative histological assessment of intestinal segments showed significant gut injury in the FF group compared to the BO and FO groups (Fig 2D, ** p<0.01 vs. FF). Although not specific for NEC, villus length was significantly decreased in both the FF and BO groups (Fig 2E), but the FF group was more severely affected.

**Fig 3:**
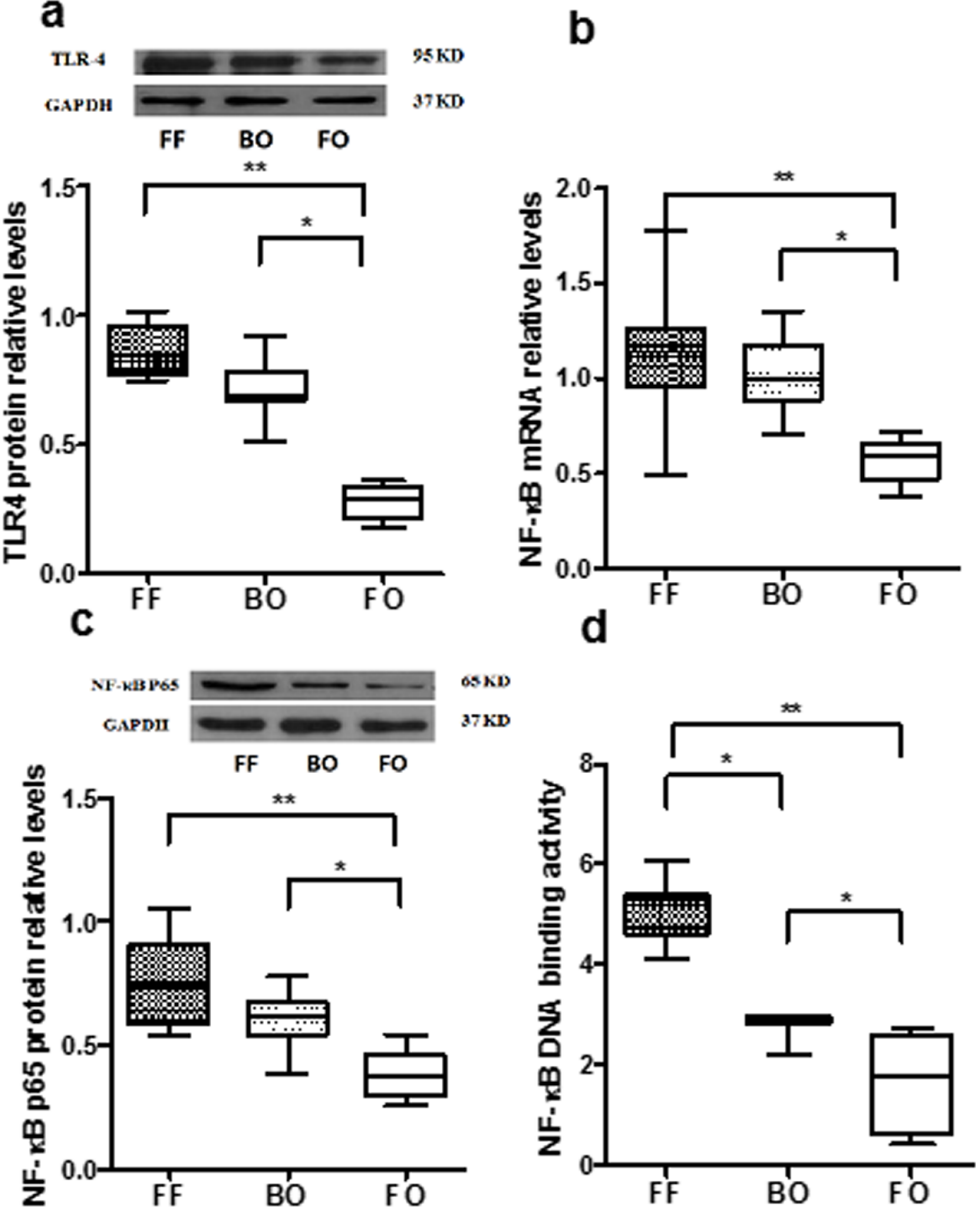
p65 NF-κB and TLR4 activation and expression. Intestinal p65 NF-κB activation in each group was assayed by EMSA. NF-κB mRNA was determined by qRT-PCR. P65 NF-κB and TLR4 proteins were measured by Western blot. Fig 3A: Image and bar graphs showed TLR4 protein expressions. Significant increase in TLR4 protein were seen in FF and BO groups. Fig 3B: Intestinal NF-κB mRNA expression levels. NF-κB mRNA levels in FF group were higher than BO and FO groups. Fig 3C: Intestinal p65 NF-κB protein levels. The levels of p65 NF-κB protein from FF group were higher than BO and FO groups. Fig 3D: Bar graphs, obtained by densitometric analysis of EMSA (the images not shown). The DNA binding activities in FF and BO groups were significantly increased compared to FO group. The data are represented as mean ± SE (* P<0.05, **P<0.01, N = 5–8 / group).

Molecular analysis of inflammation was performed by measuring the relative abundance of TLR4, p65 NF-κB protein, NF-κB activation and inflammatory cytokine (IL-1β and TNF-α) levels in intestinal segments from each group. The levels of TLR4 protein were significantly increased in the FF and BO groups (Fig 3A) compared to FO. Consistent with this observation, p65 NF-κB mRNA (Fig 3B) and protein (Fig 3C) expression were also increased in the FF and BO groups (p<0.05 or P<0.01 vs FO). While NF-κB activation (Fig 3D) was elevated in both the FF and BO, it was significantly higher in the FF group. These data demonstrate introduction of fermented formula into piglet intestines enhance activation of the NF-κB inflammatory pathway.

**Fig 4:**
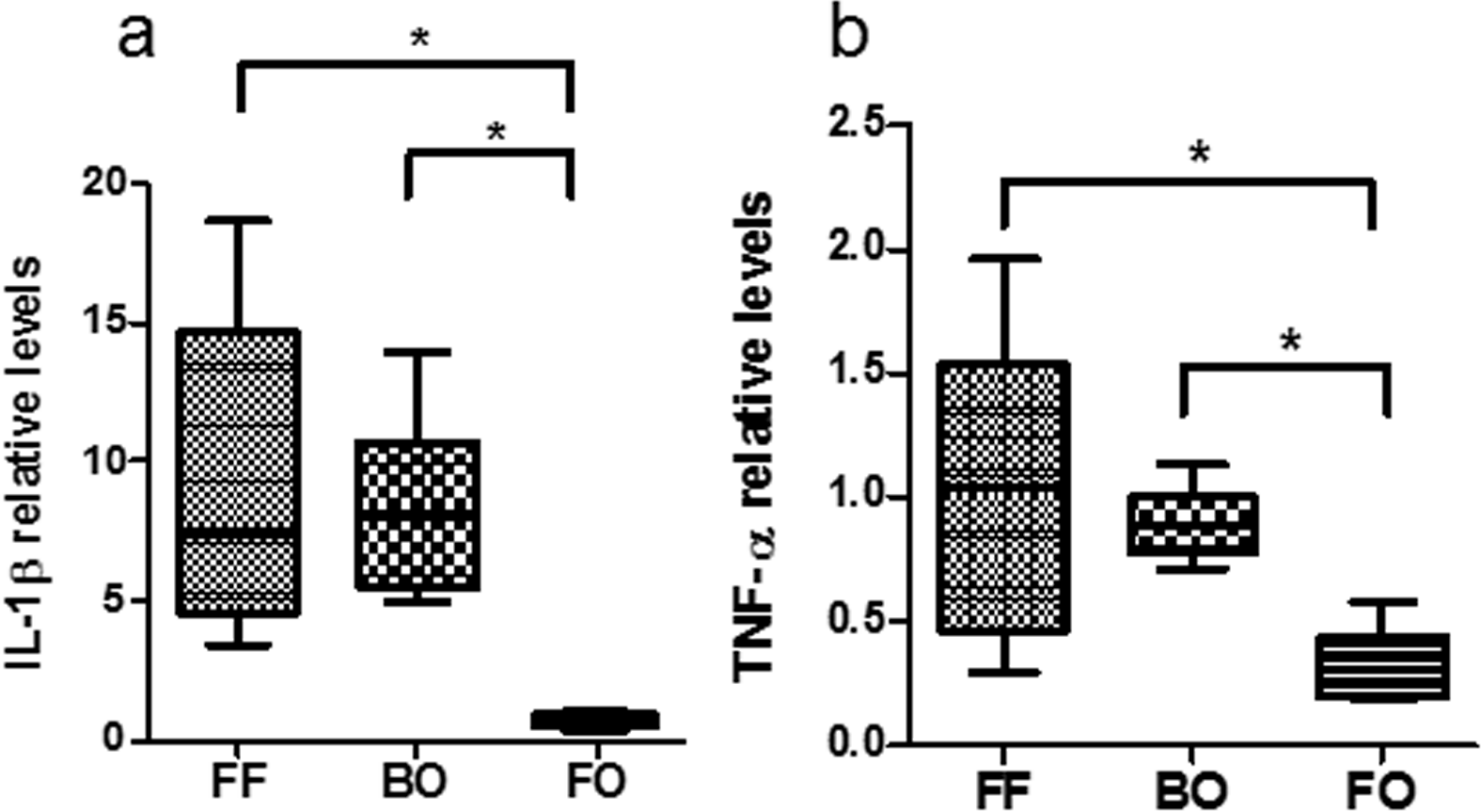
Intestinal cytokine expressions. The relative abundance of inflammatory cytokines IL-1β (Fig 4A) and TNF-α (Fig 4B) were assayed by ELISA. Total small intestinal cell lysates were extracted from three groups (FF, BO and FO). The levels of IL-1β and TNF-α were significantly increased in FF and BO groups compared to FO group. The data are expressed by fold induction normalized to total protein and expressed as mean ± SE (* P<0.05, ** P<0.01, N = 5–8 / group).

One consequence of NF-κB activation is the transcription of inflammatory cytokines TNF-α and IL-1β. The relative abundance of IL-1β and TNF-α were measured in intestinal segments from the different experimental groups. Significantly increased levels of IL-1β (Fig 4A) and TNF-α (Fig 4B) were observed in both the FF and BO groups (p<0.01 vs FO).

**Fig 5:**
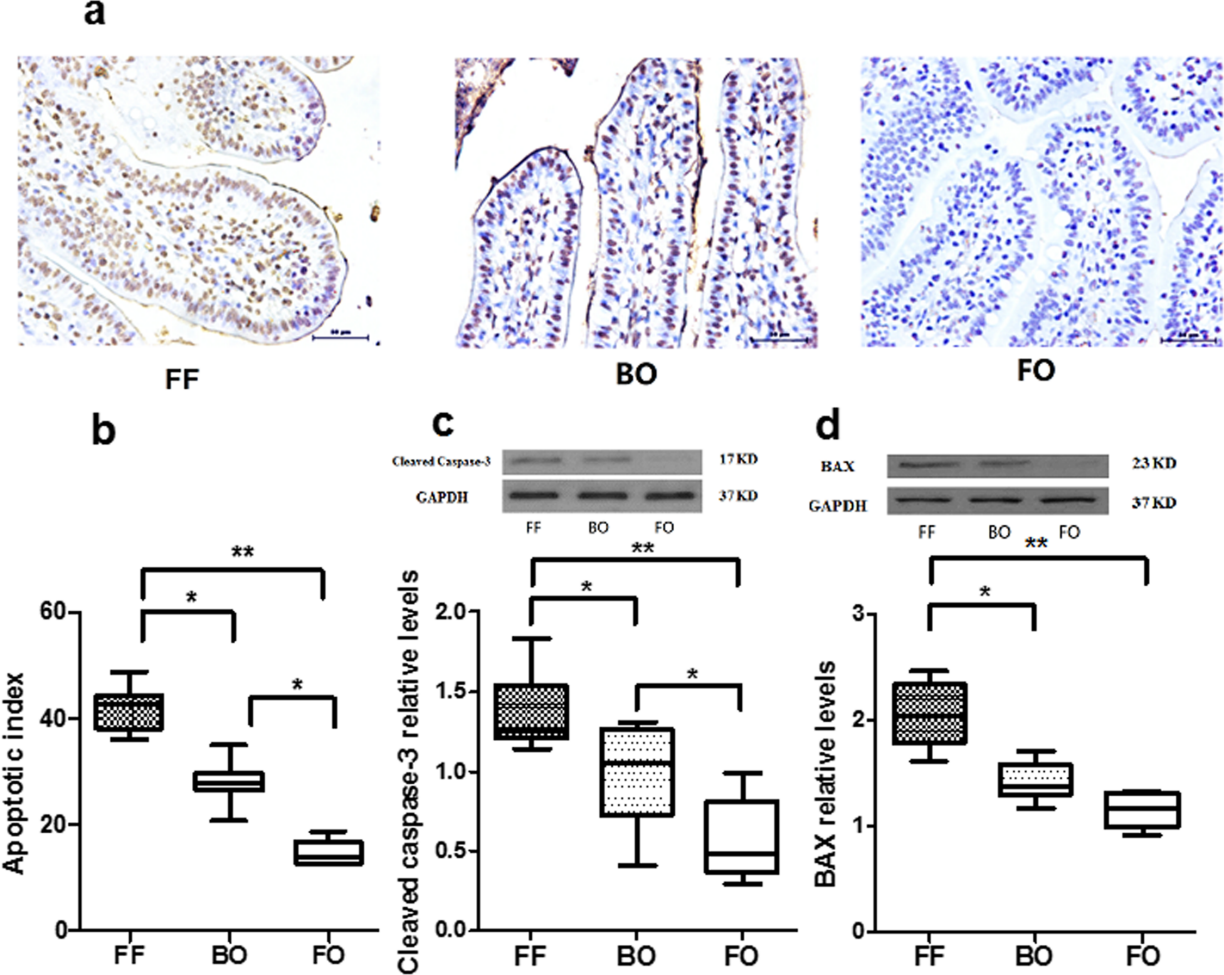
Induction of apoptosis. **Fig 5A and 5B**: Apoptotic index: Apoptotic cells were determined by TUNEL. Representative TUNEL-positive apoptotic cell is exhibited by dark brown color (Fig 5A). Three images represent different treatment in newborn piglets. The percentages of apoptotic cells in each group are presented in bar graph from quantification of images (Fig 5B). **Fig 5C and 5D**: Pro-apoptotic proteins: Intestinal cleaved caspase-3 (Fig 5C) and BAX (Fig 5D) protein expressions in each group were determined by Western blot. Cleaved caspase-3 levels in FF and BO groups were significantly increased compared to FO group. Higher levels of cleaved caspase-3 in FF group were noticed compared to BO group. BAX protein in FF group significantly increased compared to BO and FO groups. The data are represented as mean ± SE (* P<0.05, **P<0.01, N = 5–8 / group).

Apoptosis or programmed cell death of the intestinal epithelia is another characteristic of NEC. Immunohistochemical analysis of intestinal segments from the different groups were performed to assess apoptotic index. Cleaved caspase-3 and Bax protein levels were measured to assess the pro-apoptotic environment. As shown in Fig 5A and 5B, the apoptotic index was increased in small bowel from the BO and FF groups compared to FO and significantly increased in the FF group compared with Bacteria alone. These findings are consistent with similar changes in the pro-apoptotic protein cleaved caspase-3 (Fig 5C) and BAX (Fig 5D). Apoptosis was induced in FF and BO groups (vs. FO, P<0.05 or P<0.01) and higher induction of apoptosis was shown in FF group (vs. BO, P<0.05).

**Fig 6:**
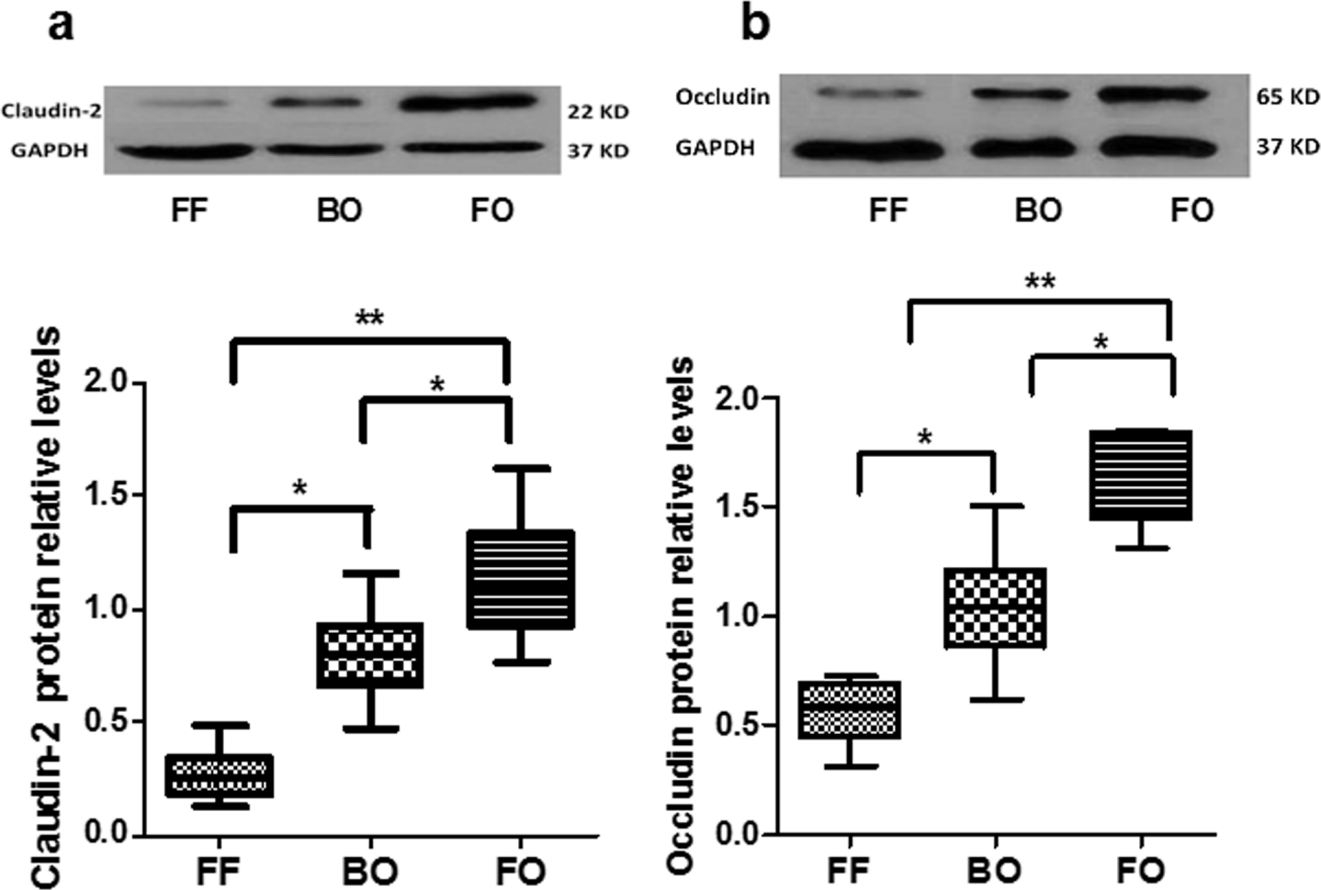
Claudin-2 and occludin protein expressions. Western blot was used to measure claudin-2 (Fig 6A) and occludin (Fig 6B) protein expressions in intestine. The levels of claudin-2 and occludin in FF and BO groups were significantly decreased compared to the FO group. Claudin-2 and occludin levels were also lower in the FF group compared to the BO group. The data are presented as mean ± SE (* P<0.05, **p<0.01, N = 5–8 / group).

The relative abundance of intercellular tight junction proteins claudin-2 and occludin were assessed as surrogate markers of intestinal permeability and barrier function in small bowel from the different groups. Reduced levels of claudin-2 (Fig 6A) and occludin (Fig 6B) were seen in BO and FF intestine with significantly lower levels in the FF group compared with Bacteria alone.

**Fig 7:**
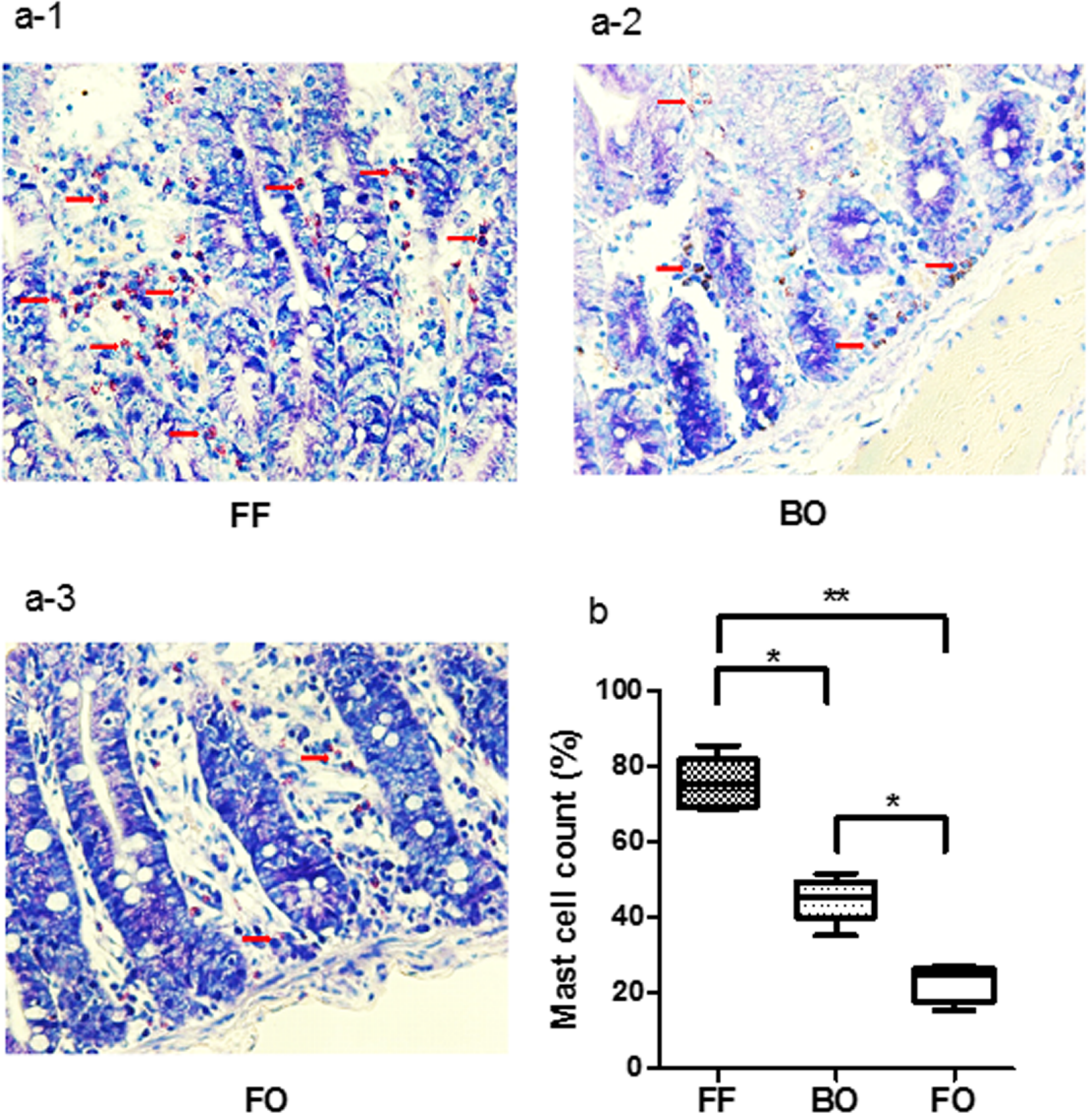
The activation of mast cells. Giemsa staining was used to examine activated mast cells in FF (Fig 7 A1), BO (Fig 7 A2) and FO (Fig 7 A3) groups. The positive standing is purple violet. The activated mast cells were displayed in the images with showing secretory granules released into the surroundings. The number of activated mast cells was counted in the villi of intestinal tissue specimens from each group. The percentage of activated mast cells over total mast cells was calculated to quantify mast cell activation (Fig 7B). The mast cell activation in FF and BO groups were shown compared to FO groups. There were significant higher activation in FF group. The data are represented as median and IQR (* P<0.05, **p<0.01, N = 5–8 / group).

The number of activated mast cells were quantified and significantly increased in the FF and BO groups (Fig 7: FF: 75.24%; BO: 41.14%; FO: 24.88%, P<0.01 vs FO).

**Fig 8:**
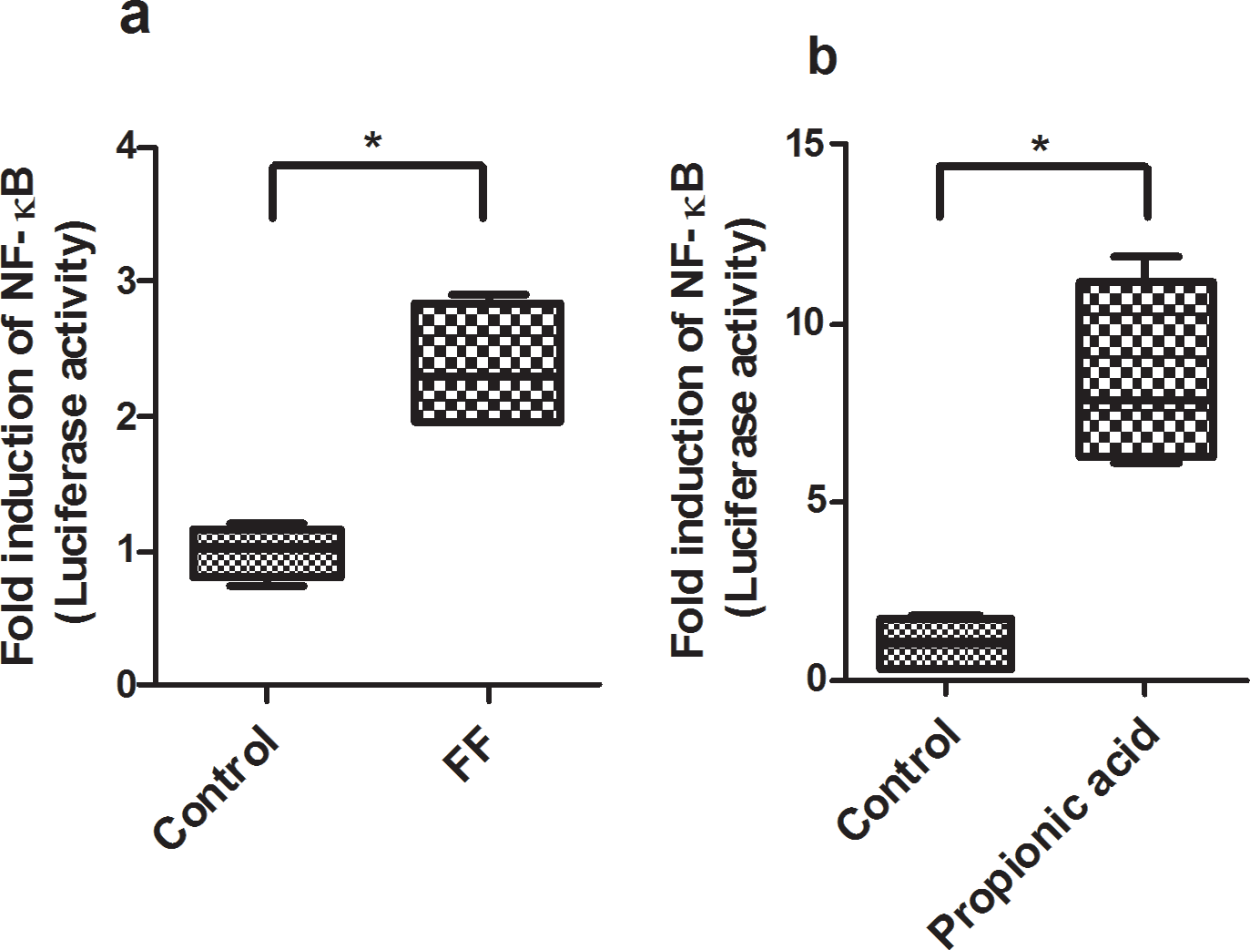
The activation of NF-κB in FHs 74 Int cells. FHS 74 int cells were transfected with pNF-κB-Luc vector, then treated with filtered fermented formula (100 μl/ml) or propionic acid (2.5 mM) in serum-free media for 18 h. Cells were harvested for luciferase activity. NF-κB luciferase activity data are expressed as fold induction of normalized luciferase activity. The levels of NF-κB luciferase activity in FF and propionic acid groups were significantly increased compared to control group. The data are represented as mean ± SE (* P<0.05, **P<0.01, N = 4 / group).

Finally, we investigated the direct effects of fermented formula and propionic acid on epithelial inflammation using the fetal intestinal cell line (FHs 74 int). As shown in Fig 8, filtered fermented formula (Fig 8A) and propionic acid (Fig 8B) both result in increased NF-κB luciferase activity compared to control groups. These data provide evidence SCFAs and FF directly regulated pro-inflammatory signaling in enterocyte cultures.

## Discussion

Our results provide evidence bacterial fermentation of formula is important in the pathogenesis of NEC. We postulate elevated levels of acetic and propionic acid in fermented formula contribute to intestinal inflammation, systemic acidosis and recapitulating clinical NEC. To our knowledge this is the first experimental model of NEC which reproduces all of the clinical characteristics of the disease including: metabolic acidosis, hemorrhagic inflammation of the bowel, transmural necrosis, intestinal perforation, pneumatosis intestinalis and portal venous gas [4, 7, 14]. Our model demonstrates the clinical associations of NEC (prematurity and enteral formula feeding) and recapitulates the histopathologic (mucosal edema, epithelial sloughing) and molecular (increased TLR4 expression, NF-κB activation, IL-1β and TNF-α) features of the disease as well.

An effective control can minimize the effects of variables other than the independent variable and increases the reliability of the results. In order to better characterize NEC model, two controls, formula (formula alone without bacterial colonization) and bacteria (bacterial colonization and “overgrowth”), were employed in our study to validate the role of fermented formula in the onset of NEC. A mix of pure bacteria and fresh formula cannot acts as a control because the fermentation will occur to produce SCFAs after introduction into piglet. Also previous study has been shown that branched chain fatty acids cannot induce complete NEC model in rats [34].

Formula alone caused no gross or molecular bowel inflammation. In contrast, *E*. *coli* alone had no significant effect on gross intestinal appearance, yet resulted in significant histopathologic and molecular evidence of inflammation. This suggests *E*. *coli* alone are insufficient to cause NEC and is consistent with the observation that although many different bacteria have been implicated in the pathogenesis of NEC, no one single microbe has been identified as causal [2, 3, 16, 17, 35, 36]. Although E. coli alone are insufficient to cause NEC, gut bacteria are likely still necessary [9]. Microbial diversity is protective against NEC [9]. Many infants admitted to the NICU are exposed to antibiotics which may reduce bacteria diversity and select for a homogenous population of bacteria with the capacity for rapid carbohydrate fermentation [35] which, when combined with formula-feeding, creates the pathogenic milieu leading to NEC.

The Bacteria group, in which the formula and fermentation products were centrifuged off, did not develop the disease and only showed mild injury by bacterial invasion. However, bacteria fermentation of cow's milk protein-based formula was sufficient to produce experimental NEC. Formula feeding has long been associated with the development of NEC, though only a fraction of formula fed NICU patients develop the disease [4, 14]. Breast fed babies have a low rate of NEC and using banked breast milk further reduces the incidence of NEC [37, 38]. Lactose, the primary carbohydrate in standard infant formula (and breast milk) is also the primary substrate for bacterial fermentation. Bacterial fermentation of lactose commonly produces SCFAs depending on the metabolic repertoire of the microflora [14, 35]. SCFAs have been shown to be highly injurious to the premature small intestine [39, 40], with propionic acidemia having been specifically implicated in NEC pathogenesis [41]. These SCFAs are a challenge for the biochemical machinery of the premature infant because they cannot enter the tricarboxylic acid cycle to be metabolized and must therefore be handled in the liver [14]. We postulate these SCFAs bypass the premature liver which lacks mature enzymes to metabolize SCFAs through Kreb’s cycle and maintained in the systemic circulation resulting in severe metabolic acidosis.

The association of prematurity with NEC is well established and increases both the risk and the severity of NEC [2]. Specific features of the premature bowel that predispose to NEC include impaired motility, immature brush border enzymes, impaired goblet cell function resulting in an attenuated mucus layer and disordered mucosal innate immune response [42]. Motility is a protective feature of the mature bowel, as irritating or pro-inflammatory luminal contents may be rapidly propelled out of the gastrointestinal tract. The hypomotility of the premature infant allows for stasis resulting in prolonged interaction of gastrointestinal flora with enteral feeds as well as prolonged contact of the mucosa with potentially injurious byproducts of this interaction. Brush border enzymes beta-galactosidase (lactase) and alpha-glucosidases (sucrase, isomaltase, maltase, and glucoamylase) reach functional maturity at different gestational stages with lactase maturing the last between 30–34 weeks [43, 44]. As the premature infant bowel is exposed to lactose-containing cow’s milk-based formula, the limited ability of the neonate’s small bowel to digest lactose provides the proximal colonic flora extra substrate to ferment. Increased availability of substrate would result in the preferential production of luminal SCFAs and the progression to NEC.

Many pro-inflammatory cytokines and chemokines are upregulated during inflammation through the activation of the transcription factor NF-κB and Toll-like receptors (TLR) [45–47]. TLR4 is a pattern recognition receptor that activates the innate immune system by NF-κB transduction followed by inflammatory cytokine production via an intracellular signaling pathway. Increasing evidence suggests that TLR4 is upregulated in premature intestine as compared with full-term intestine [48]. Activation of TLR4 on the lining of the premature intestine by the Gram-negative bacteria that colonize the premature gut results in a number of deleterious effects, including increased enterocyte apoptosis, impaired mucosal healing and enhanced pro-inflammatory cytokine release, which in aggregate lead to the development of NEC [46]. Intestinal epithelial cell inflammation is a characteristic of NEC. In the current study, increases in intestinal inflammatory cytokines (IL-1β and TNF-α), NF-κB activation and TLR4 expression were seen in both FF and BO groups, supporting the hypothesis bacteria and bacterial fermentation products cause inflammation. These findings are supported by our cell culture data which show that FF and SCFAs stimulated p65 NF-κB protein expressions which suggested that increased inflammatory signaling resulted from elevated levels of SCFAs. Characterizing the role of the innate immune system in the present model represents an important research goal. The model presented in the current study provides an excellent platform from which to study the role of these host immune responses, other cellular mechanisms, as well as therapeutic strategies for NEC.

Maintaining intestinal barrier function, protecting against inflammatory cytokines and decreasing apoptosis can lead to a decrease in the incidence of NEC in animal models [49]. Disruption of the intestinal barrier in NEC can lead to bacterial translocation, increasing inflammatory stimuli and thus intestinal injury [50]. The tight junctions between epithelial cells are formed by transmembrane spanning proteins occludin and claudin that maintain the function of the intestinal barrier [51]. In our study, levels of occludin and claudin-2 were reduced in the FF and BO groups, consistent with previous patient and animal models of NEC [5, 52]. Apoptosis was increased in the FF and BO groups, as demonstrated by increased apoptotic indices, cleaved caspase-3 and BAX protein levels. The FF group had more severe reductions in tight junction proteins and pro-apoptotic indicators compared to the Bacteria group, providing evidence significant disruption of the intestinal barrier is present in our NEC model.

Mast cells represent the first line of cellular defense against harmful antigens. They express TLRs and participate in pro-inflammatory responses through recruitment and degranulation [53]. In the setting of inflammation, such as SCFA interaction with the intestinal mucosa and breakdown of the intestinal barrier, mast cells are recruited to the mucosal surfaces [53]. The mast cells are therefore not only exposed to the inflammatory milieu brought by the fermented formula, but also to casein, the bovine protein present in most infant formulas that is viewed as antigenic by the exposed mast cells. Miller et al. demonstrated exposure of mast cells to luminal casein or casein hydrolysate in the setting of enhanced epithelial permeability leads to mast cell activation [54]. Our study shows mast cell activation was increased in the FF group, suggesting their potential involvement in the inflammation and loss of intestinal barrier function in NEC.

Our results suggest bacteria alone cannot induce NEC. However, administration of FF reproduced the gross, histologic and molecular phenotype of human NEC. This is likely due to the pathogenesis of NEC being multifactorial, involving bacterial presence, intestinal hypomotility, the acidic environment created by the SCFAs, loss of intestinal barrier function, and innate immune system activation by antigenic casein.

## Limitations

The piglets used in our study were not premature, but exhibit all the classic features of NEC. In human neonates, NEC symptoms may take days to weeks to manifest which may be due to the time required for the microbiome to change following antibiotic administration and selection pressure of luminal formula. Another potential limitation is that only female piglets were used in this study and additional studies will need to be performed in male piglets, though gender has not been identified as a risk factor of NEC.

In our study, the *E*. *coli* were mixed with formula and incubated for 24 hours such that fermentation products were already present when injected into the intestine resulting in the rapid progression of NEC (4–6 hours). While the FF group demonstrated increased levels of SCFAs and incubation of enterocytes with propionic acid caused NF-κB activation, it is also possible that other products of fermentation contribute to gut injury in this model. The ex vivo fermentation of formula is also a limitation of our study design and the levels of SCFAs measured in our study may be different *in vivo*. A more clinically relevant model might be achieved by allowing the fermentation process to occur *in vivo*.

## Conclusions

Our hypothesis for the pathogenesis of NEC is based on clinical associations of the disease. Our results provide evidence fermentation of formula by *E*. *coli* generates SCFAs which when fed to newborn piglets reproduces the phenotype of neonatal NEC. Of note, *E*. *coli* alone caused histologic and molecular inflammation. While other large animal models have reported pneumatosis intestinalis, they have not demonstrated portal venous gas [33]. To our knowledge this is the first animal model of NEC to reproduce the finding of portal venous gas, a hallmark of human NEC. Of note, the NEC phenotype was created in this animal model without exposure to systemic stressors like asphyxia-hypoperfusion or hypothermia.

## Supporting information

S1 Table Supporting data file. Summarizing and analyzing the experimental data.
(XLSX)
